# Telomerase Activation to Reverse Immunosenescence in Elderly Patients With Acute Coronary Syndrome: Protocol for a Randomized Pilot Trial

**DOI:** 10.2196/19456

**Published:** 2020-09-23

**Authors:** Rebecca Maier, Bilal Bawamia, Karim Bennaceur, Sarah Dunn, Leanne Marsay, Roland Amoah, Adetayo Kasim, Andrew Filby, David Austin, Helen Hancock, Ioakim Spyridopoulos

**Affiliations:** 1 Newcastle Clinical Trials Unit Newcastle University Newcastle Upon Tyne United Kingdom; 2 James Cook University Hospital Middlesbrough United Kingdom; 3 Institute of Genetic Medicine Newcastle University Newcastle Upon Tyne United Kingdom; 4 Wolfson Research Institute for Health and Wellbeing Durham University Durham United Kingdom; 5 Flow Cytometry Core Facility Newcastle University Newcastle Upon Tyne United Kingdom

**Keywords:** coronary heart disease, acute coronary syndrome, immunosenescence, telomerase activator

## Abstract

**Background:**

Inflammation plays a key role in the pathophysiology of coronary heart disease (CHD) and its acute manifestation, acute coronary syndrome (ACS). Aging is associated with a decline of the immune system, a process known as immunosenescence. This is characterized by an increase in highly proinflammatory T cells that are involved in CHD progression, plaque destabilization, and myocardial ischemia–reperfusion injury. Telomere dysfunction has been implicated in immunosenescence of T lymphocytes. Telomerase is the enzyme responsible for maintaining telomeres during cell divisions. It has a protective effect on cells under oxidative stress and helps regulate flow-mediated dilation in microvasculature.

**Objective:**

The TACTIC (Telomerase ACTivator to reverse Immunosenescence in Acute Coronary Syndrome) trial will investigate whether a telomerase activator, TA-65MD, can reduce the proportion of senescent T cells in patients with ACS with confirmed CHD. It will also assess the effect of TA-65MD on decreasing telomere shortening, reducing oxidative stress, and improving endothelial function.

**Methods:**

The study was designed as a single-center, randomized, double-blind, parallel-group, placebo-controlled phase II trial. Recruitment started in January 2019. A total of 90 patients, aged 65 years or older, with treated ACS who have had CHD confirmed by angiography will be enrolled. They will be randomized to one of two groups: TA-65MD oral therapy (8 mg twice daily) or placebo taken for 12 months. The primary outcome is the effect on immunosenescence determined by a decrease in the proportion of CD8+ TEMRA (T effector memory cells re-expressing CD45RA [CD45 expressing exon A]) cells at 12 months. Secondary outcomes include leukocyte telomere length, endothelial function, cardiac function as measured by echocardiography and NT-proBNP (N-terminal fragment of the prohormone brain-type natriuretic peptide), systemic inflammation, oxidative stress, and telomerase activity.

**Results:**

The study received National Health Service (NHS) ethics approval on August 9, 2018; Medicines and Healthcare products Regulatory Agency approval on October 19, 2018; and NHS Health Research Authority approval on October 22, 2018. The trial began recruiting participants in January 2019 and completed recruitment in March 2020; the trial is due to report results in 2021.

**Conclusions:**

This pilot trial in older patients with CHD will explore outcomes not previously investigated outside in vitro or preclinical models. The robust design ensures that bias has been minimized. Should the results indicate reduced frequency of immunosenescent CD8+ T cells as well as improvements in telomere length and endothelial function, we will plan a larger, multicenter trial in patients to determine if TA-65MD is beneficial in the treatment of CHD in elderly patients.

**Trial Registration:**

ISRCTN Registry ISRCTN16613292; http://www.isrctn.com/ISRCTN16613292 and European Union Drug Regulating Authorities Clinical Trials Database (EudraCT), European Union Clinical Trials Register 2017-002876-26; https://tinyurl.com/y4m2so8g

**International Registered Report Identifier (IRRID):**

DERR1-10.2196/19456

## Introduction

### Background

Inflammation plays a key part in the pathophysiology of coronary heart disease (CHD) and its acute manifestation—acute coronary syndrome (ACS)—from atheroma formation to plaque rupture [1]. Despite contemporary treatment, recurrent adverse events (AEs) post-ACS remain common, especially in the elderly [2]. Aging is associated with a decline of the immune system, a process known as immunosenescence [3], leading to an increased burden of disease. Targeting interleukin 1 (IL-1β), the principal cytokine of innate immunity, in patients with previous myocardial infarction (MI) has been shown to reduce subsequent adverse cardiovascular outcomes [4]. Furthermore, the contribution of the adaptive immune system to the complex inflammatory response evoked during ACS has been demonstrated extensively in experimental and clinical studies [5]. In particular, highly proinflammatory senescent T cells are thought to be key players in plaque destabilization [6] and myocardial ischemia–reperfusion injury [7].

Telomere dysfunction has been implicated in immunosenescence of T cells [3,8]. Telomeres are DNA caps that protect the ends of chromosomal DNA. They are widely regarded as the internal biological clock of a living organism and shorten by a few base pairs with every cell division. This process can be slowed down by activation of telomerase, which is responsible for producing and maintaining telomeres. Shorter lymphocyte telomeres are associated with development of CHD as well as increased cardiovascular risk and mortality independent of conventional vascular risk factors [9-12]. Alleles associated with shorter telomere length (TL) were also associated with an increased risk of CHD, suggesting a causal relationship [13].

The TACTIC (Telomerase ACTivator to reverse Immunosenescence in Acute Coronary Syndrome) trial was designed to test whether a telomerase activator can reduce the proportion of senescent T cells in patients following ACS. There is accumulating evidence that telomerase, through its telomerase reverse transcriptase (TERT) catalytic subunit, contributes to cell physiology independently of its ability to elongate telomeres. We and our collaborators have demonstrated the effect of oxidative stress on telomerase as well as a telomere-independent protective effect of telomerase in cells under oxidative stress [14,15]. Telomerase activity (TA) has also been shown to regulate endothelial flow-mediated dilation (FMD) [16].

The small molecule cycloastragenol (CAG), isolated from the roots of the herb astragalus, is the only available telomerase activator in humans. TA-65MD is a purified and encapsulated form of CAG with increased bioavailability (T.A. Sciences). We have also shown that TA-65MD induces telomerase and proliferation in CD4 T lymphocytes in a TERT-dependent way [17]. In a randomized controlled trial investigating cytomegalovirus (CMV)-positive healthy subjects aged 53-87 years old, subjects taking 8 mg of TA-65MD daily increased TL over the 12-month period, whereas subjects in the placebo group significantly lost TL [18].

### Trial Rationale

The evidence indicates that telomerase deficiency in atherosclerosis leads to accelerated immunosenescence with telomere shortening in peripheral blood leukocytes, increased oxidative stress and inflammation, and impaired microvascular endothelial function, all of which contribute to CHD progression. We hypothesize that activating telomerase with TA-65MD will lead to reduced immunosenescence, decreased telomere shortening, and improved endothelial function in patients with CHD. The null hypothesis is that there will be no difference in immunosenescence between the two groups following 12 months of treatment with TA-65MD or placebo. The choice of active treatment versus placebo is appropriate in this population where no telomerase activator is currently used as part of usual care. All patients will receive usual care alongside the trial.

### Objectives

#### Primary Objective

We aim to assess the effect of 8 mg of oral TA-65MD given twice daily for 12 months on immunosenescence in older patients following ACS.

#### Secondary Objectives

The secondary objectives of this trial are as follows:

1. To investigate the effect of 1-year TA-65MD treatment on leukocyte TL.
2. To investigate the effect of 1-year TA-65MD treatment on microvascular endothelial function.
3. To investigate the effect of 1-year TA-65MD treatment on systemic inflammation and heart failure, reflected by expression of N-terminal fragment of the prohormone brain-type natriuretic peptide (NT-proBNP) and high‐sensitivity C‐reactive protein (hsCRP).
4. To investigate the effect of 1-year TA-65MD treatment on measures of cardiac function as measured by echocardiography.
5. To investigate the effect of TA-65MD treatment on TA and oxidative stress.
6. To investigate the effect of TA-65MD treatment on clinical events—all-cause death, stroke, or MI—in patients after 1 year.
7. To characterize the AE profile of TA-65MD.
8. To quantify adherence to study drugs.
9. To investigate the impact of seropositivity to CMV at baseline on trial outcomes.

## Methods

### Trial Design

This is a single-center, randomized, double-blind, parallel-group, placebo-controlled phase II trial comparing TA-65MD with placebo in 90 participants with CHD who have had ACS in the 6 months prior to consent. A total of 90 patients will be randomized to either the TA-65MD group (n=45) or the placebo group (n=45); TA-65MD and placebo will be taken by these groups, respectively, twice daily for 12 months. The schedule of this trial is shown in Table 1. The trial will run according the International Conference on Harmonisation (ICH)-Good Clinical Practice (GCP) and in accordance with relevant UK legislation and the trial protocol. This trial was registered at the International Standard Randomized Controlled Trial Number (ISRCTN) registry (16613292) and at the European Union Drug Regulating Authorities Clinical Trials Database (EudraCT), European Union Clinical Trials Register (2017-002876-26).

**Table 1:** Trial schedule of assessments and interventions.

Schedule items		Time of assessments and interventions						
		Baseline^a^	Day 1	1 month (wks 3-5)	3 months (wks 11-15)	6 months (wks 24-28)	9 months (wks 37-41)	12 months (wks 50-54)
**Enrollment**								
	Eligibility screen	X						
	Informed consent	X						
**Assessments**								
	Blood pressure	X		X	X	X	X	X
	Capillary glucose	X		X	X	X	X	X
	Physical assessment of height and weight	X						
	Venous sample (5 mL) cytomegalovirus IgG^b^	X						
	Venous sample (4 mL) CD8 TEMRA^c^ immunosenescence (primary outcome)^d^	X				X		X
	Venous sample (36 mL) CD8 and CD4 immunosenescence (secondary outcomes), telomere length, and telomerase activity	X				X		X
	Venous sample (4 mL) oxidative stress, future use	X				X		X
	Venous sample (5 mL) future research use—optional consent	X				X		X
	Venous sample (5mL) hsCRP^e^ and NT-proBNP^f^	X				X		X
	Endothelial function (EndoPAT [Peripheral Arterial Tone])	X				X		X
	Echocardiography	X						X
**Interventions**								
	Randomization—stratified	X						
	Dispensing of investigational medicinal product (IMP)		X	X	X	X	X	X
	Return of unused IMP and drug adherence			X	X	X	X	X
	Adverse events evaluation			X	X	X	X	X

^a^Baseline assessments are completed after written consent is obtained and are performed before randomization.

^b^IgG: immunoglobulin G.

^c^TEMRA: T effector memory cells re-expressing CD45RA (CD45 expressing exon A).

^d^Results from baseline immunosenescence sample are required for randomization.

^e^hsCRP: high‐sensitivity C‐reactive protein.

^f^NT-proBNP: N-terminal fragment of the prohormone brain-type natriuretic peptide.

### Primary Outcome

**Figure 1:**
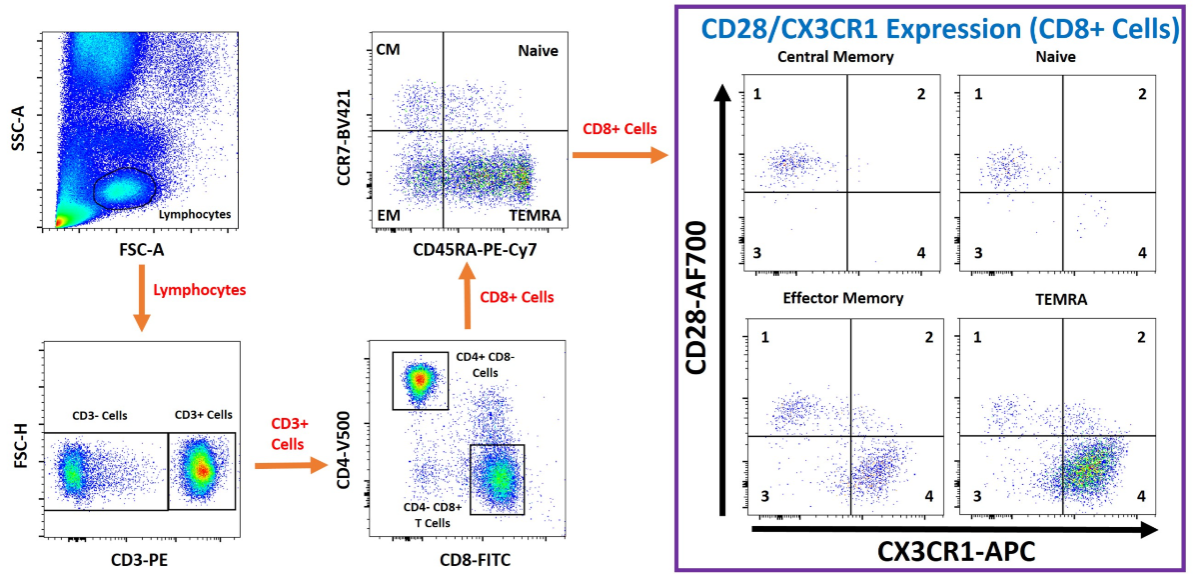
Example gating path for seven-color fluorescence-activated cell sorting (FACS) from ethylenediaminetetraacetic acid (EDTA) peripheral blood. AF700: Alexa Fluor 700; APC: allophycocyanin; BV421: Brillian Violet 421; CCR7: C-C chemokine receptor type 7; CD45RA: CD45 expressing exon A; CM: central memory; CX3CR1: C-X3-C motif chemokine receptor 1; EM: effector memory; FITC: fluorescein isothiocyanate; FSC: forward scatter; FSC-H: forward scatter-pulse height; PE: phycoerythrin; PE-Cy7: phycoerythrin and cyanine 7; SSC: side scatter; TEMRA: T effector memory cells re-expressing CD45RA; V500: violet 500.

The primary outcome is immunosenescence following 12 months of treatment with TA-65MD. Immunosenescence will be determined by flow cytometry and fluorescence-activated cell sorting (FACS) (see Figure 1); the proportion of terminally differentiated CD8+ effector memory cells (% CD8+ TEMRA [T effector memory cells re-expressing CD45RA (CD45 expressing exon A)]) will be calculated from the total number of peripheral blood CD8+ T lymphocytes. We have previously demonstrated the prognostic utility of CD8+ TEMRA as a measure for immunosenescence [19]. The mean difference between the intervention and control arms will be compared at 12 months.

### Secondary Outcomes

#### CD8 T Cell Telomere Length

TL will be determined from cryopreserved peripheral blood mononuclear cells (PBMCs) by flow cytometry-fluorescent in situ hybridization (flow-FISH) [9]. TL will be measured in total leukocytes and CD8+ T cells. CD8+ T cell TL is significantly reduced in patients with CHD and post-MI [9]. We expect TL in CD8+ T cells to decrease less in patients treated with TA-65MD. TL will be measured in total leukocytes and in CD8+ T cells at baseline, 6 months, and 12 months.

#### Microvascular Endothelial Function

Microvascular endothelial function will be assessed by measuring FMD at baseline, 6 months, and 12 months. Using plethysmography at the fingertips of both hands, the EndoPAT (Peripheral Arterial Tone) system (Itamar Medical Ltd) will calculate an index of pulse wave amplitude after cuff occlusion to before occlusion of the test arm divided by the same ratio of the control arm, namely the reactive hyperemic index. FMD has been shown to be compromised in patients with CHD and microvascular dysfunction as a predictor of adverse clinical outcome [20].

#### Systemic Inflammation

Systemic inflammation will be assessed using the hsCRP, which is a downstream biomarker of inflammation associated with an increased risk of cardiovascular events [21].

#### Cardiac Function

Cardiac function will be assessed by NT-proBNP and transthoracic echocardiography at baseline and 12 months. Together, these measures will determine myocardial function, strain (NT-proBNP), and global longitudinal strain, reflecting the pathophysiological targets of heart failure. Lack of telomerase and presence of shortened telomeres in cardiomyocytes from preclinical models (ie, mice) have been shown to be critical in the development of heart failure in that species [22]. Patients with chronic heart failure have shorter TL compared to age- and gender-matched controls with incremental attrition according to the presence and extent of coronary artery disease (CAD) [23].

#### Telomerase Activity

TA will be assessed by a modified telomeric repeat amplification protocol (TRAP) assay, using digital droplet polymerase chain reaction (PCR) [24,25]. This provides an indication of drug effect: TA-65MD is expected to increase TA in PBMCs.

#### Oxidative Stress

Oxidative stress will be measured with the thiobarbituric acid reactive substances (TBARS) colorimetric assay (Oxford Biomedical Research) from freshly collected and cryopreserved plasma. TBARS is an established assay to quantify lipid peroxides [26].

#### Seropositivity to Cytomegalovirus

The effect of CMV seropositivity at baseline will be correlated with study outcomes using an exploratory analysis. CMV is a highly prevalent human herpes virus with CMV seropositivity present in a large proportion of elderly patients. CMV-directed cells increase with age, constituting a large proportion of the total CD8+ T cell pool in elderly individuals [27]. T cell responses to CMV are restricted to a limited number of epitopes, resulting in progressive, prolonged oligoclonal expansion of CMV-specific CD8+ T cells in a process known as memory inflation [28]. We have previously shown that individuals who are seropositive have a much higher proportion of CD8+ TEMRA cells and have a greater risk of heart attacks [7,29].

#### Clinical Outcomes and Adverse Events

Major clinical events will be measured for 12 months from the start of treatment. All-cause death, MI, and stroke will be presented as a composite outcome—major adverse cardiac and cerebrovascular events (MACCE)—and also reported separately. All AEs, including those considered related and unrelated to the intervention, will be recorded and reported from the time of randomization for 12 months or until a patient has completed all trial activities.

### Study Setting

The trial aims to recruit 90 patients. All patients will be recruited, treated, and followed up at a single site: The James Cook University Hospital, Middlesbrough, UK. Blood samples will be analyzed at site laboratories and, where specialist equipment and expertise are required, will be transported to the Institute of Genetic Medicine, Newcastle University, UK, for analysis.

### Study Visits and Assessments

Following baseline assessment and initiation of treatment—placebo or TA-65MD—patients in both groups will be followed up at 1 month, 3 months, 6 months, 9 months, and 12 months (see Table 1). At each visit, drug adherence will be assessed and the patient will be evaluated for AEs.

### Inclusion and Exclusion Criteria

Textbox 1 lists the criteria informing the eligibility or exclusion of patients for the trial.

**Inclusion and exclusion criteria.**

Inclusion criteria—patients will be eligible for the trial if they:

- Provide written informed consent
- Are male or female, aged 65 years or over, with an index presentation of an acute coronary syndrome (ACS)* within the previous 6 months
- Have successfully completed revascularization** or are being managed medically following ACS
- Have angiographic evidence of coronary heart disease: at least one major epicardial vessel stenosis ≥70%
- Are recruited more than 24 hours after presentation with the index ACS event

*ACS defined as either a non-ST elevation myocardial infarction (NSTEMI) or ST elevation myocardial infarction (STEMI)

**Percutaneous coronary intervention or angioplasty, eligible the following day, or coronary artery bypass grafting, eligible 3 months following surgery

Exclusion criteria—patients will be excluded from the trial if they:

- Have any disorder associated with immunological dysfunction (ie, acute or chronic inflammatory or neoplastic coexisting disease, known positive serology for HIV, or hepatitis)
- Are clinically unstable (ie, hemodynamically unstable, cardiogenic shock, or unconscious)
- Have severe, uncontrolled hypertension (blood pressure [BP] >170/110 mmHg or ambulatory BP of 150/95 mmHg)
- Have a severe comorbidity that has impact on outcome over the next 2 years
- Are taking immunosuppressants
- Have a known malignancy
- Currently use a nutritional supplement derived from the roots of the
  Astragalus
  species
- Have a previous known substance addiction
- Have insulin-dependent diabetes
- Are judged by the investigator that they should not participate in the study, for example, on the basis of previous serious psychiatric illness or are unlikely to comply with study procedures, restrictions, and requirements
- Have participated in any other interventional medicinal study in the past 6 months

### Trial Procedures

#### Screening, Recruitment, and Consent of Participants

Potential participants will be identified by the clinical team following admission to hospital with ACS and having agreed to undergo coronary angiography or having already had an angiography that confirms CHD. Patients may also be identified following discharge. Potentially eligible participants will be invited to participate by a delegated member of the study team. The trial will be explained by the clinical research team and, after time for questions, written consent will be sought from the patient. Written informed consent will be obtained prior to any trial specific procedures and prior to randomization. There will be additional consent for storage of blood samples for up to 5 years for future use in ancillary studies. Patients have the right to withdraw from the trial at any time and without providing a reason. With the explicit consent of patients, general practitioners will be informed of their participation.

#### Eligibility Assessment

Following consent, the principal investigator or a subinvestigator on the delegation log will confirm the patient’s eligibility. Patients who do not meet trial eligibility criteria prior to randomization will be considered a *screen failure* and withdrawn from the trial with no further data collected.

#### Randomization

Randomization will be performed using the Sealed Envelope Ltd system with a minimization scheme to ensure patients randomized to each group are comparable at baseline. The minimization scheme will account for (1) gender (male or female), (2) type of ACS (ST elevation myocardial infarction [STEMI] or non-ST elevation myocardial infarction [NSTEMI]), and (3) CD8+ TEMRA (high >45% or low ≤45%) at baseline.

Eligible patients will be randomized by delegated and trained members of the research team at each center using the 24-hour, central, secure, web-based randomization system with concealed allocation. Eligible patients will be randomized in a 1:1 ratio to receive TA-65MD (intervention under study) or placebo (control arm).

#### Blinding

Assignment to either the TA-65MD or placebo groups will be blinded to the patient, treating clinicians, and the clinical research team, including the pharmacy, research nurses, echocardiogram assessors, laboratory staff, and the chief investigator. Blinding will be maintained in the randomization system, the electronic case report forms (eCRFs), and on investigational medicinal product (IMP) labels. All members of the Newcastle Clinical Trial Unit (NCTU) and the statistics team will be blinded, with the exception of the trial data manager to enable reporting to the independent data monitoring committee as appropriate. The IMP will be labeled using a unique identification code, which will be linked to the study randomization system. TA-65MD and its matched placebo will be identical and presented in the same packaging to ensure blinding of the IMP.

#### Unblinding

Unblinding should not occur except in the case of medical emergencies, where the appropriate management of the patient requires the knowledge of the randomization allocation. To avoid unnecessary unblinding, it will be assumed that the patient is on active treatment within the trial. The Sealed Envelope Ltd online web-based randomization service will be used for emergency unblinding. The primary trial analysis will be performed prior to unblinding. All patients will be informed of which arm they were assigned to, once analysis of the trial data is complete.

### Study Intervention

#### Intervention Under Study: TA-65MD

TA-65MD is marketed as a dietary supplement. The active ingredient of TA-65MD is 1.8% CAG, isolated from roots of the *Astragalus* species. The active ingredient has been identified in an empirical screen of traditional Chinese medicine plant extracts and compounds. TA-65MD is an activator of telomerase, an enzyme whose actions protect the ends of chromosomes from shortening associated with repeated cellular replication. Patients allocated to the intervention will be given 8 mg of TA-65MD twice daily.

#### Control Arm: Placebo

The matched placebo has been manufactured to ensure that it is consistent with TA-65MD in appearance, taste and smell, labeling, packaging, and batch number. Patients allocated to the placebo group will be given this twice daily.

### Safety Data

#### Overview

An independent expert panel has determined TA-65MD to be Generally Recognized As Safe (GRAS) for use in a medical food under the provisions of the Federal Food, Drug, and Cosmetic Act, administered by the US Food and Drug Administration. T.A. Sciences, Inc, provided extensive animal and human clinical data to support the status of GRAS. The safety of TA-65MD has been assessed in an observational study of 114 adults over the course of 1 year [30]. At doses up to 50 mg per day there were no AEs reported for TA-65MD. Safety was also assessed in a randomized, placebo-controlled study involving 117 adult volunteers over the course of 1 year, with no toxicities detected in the liver, kidneys, and metabolic functions as assessed by biochemical markers [29]. An anticipated risk for the use of TA-65MD relates to its ability to activate telomerase. This is the subject of scientific conjecture in consideration of its relationship to cancer [31,32]. An in vitro assay testing the telomerase-activating potency of TA-65 (ie, CAG not in capsules) in Medical Research Council cell strain-5 (MRC-5) fibroblasts suggested there was dose-related TA (Sierra Sciences Labs). While telomerase activation has been observed, TA-65MD does not increase the lifespan of cells. In fact, the preliminary observation of a controlled in vivo cancer study using 5 mL/kg of TA-65 (ie, CAG not in capsules) or sham drug administered by oral gavage for up to 40 days in mice xenografted with four different human tumors—lung (H460), colon (HT29), breast (MDA-MB-435), and prostate (PC3)—suggests a trend toward tumor growth retardation in two cancer types; as well, there was no statistically significant adverse effects on body weight or tumor size for any of the cell lines nor on the growth rate of the tumors (internal T.A. Sciences document, 2008). Given that the population under study in the TACTIC trial are elderly with ACS, and that there were no data on the impact of the drug on the outcomes or population, we chose to conduct the trial using a conservative dose of 8 mg twice daily.

#### Known Side Effects

TA-65MD may interfere with medications that suppress the immune system and may also affect blood sugar and blood pressure. In an observational study, two subjects self-reported an “anxious” feeling shortly after voluntarily increasing their daily consumption of TA-65MD to 100 mg/day—a self-imposed choice without physician approval at a consumption level two times above the intended dose for this observational study; the feelings resolved in both subjects when daily consumption was returned to 50 mg/day.

### Administration and Adherence

Participants may begin taking the IMP immediately following the completion of all baseline assessments, confirmation of eligibility, and randomization. A dose of 8 mg of TA-65MD (T.A. Sciences) or matched placebo will be taken as 1 × 8-mg capsule twice daily (ie, morning and evening). Participants will take the allocated IMP until the end of follow-up at 12 months. Patients will be prescribed IMP following randomization and at 1, 3, 6, and 9 months following the start of the intervention. Returned capsules at each visit will be used to calculate the adherence for each participant. Concomitant medications will also be reviewed and documented at each visit.

### Withdrawal of Participants

Participants will be made aware of their right to withdraw from the trial at any time for any reason and without giving a reason. Participants will be withdrawn from the trial by the clinical team for any of the following reasons:

1. Intercurrent illness means the participant is no longer able to complete study procedures.
2. The patient suffers unacceptable side effects caused by the study drug.
3. Suspected unexpected serious adverse reactions occur.

The investigator can withdraw participants in the event of any reason that would compromise participant safety or the validity of the results. Data and blood samples collected up to the point of withdrawal will be kept and used in the analysis of the trial unless the participant explicitly requests for these to be removed.

### Pharmacovigilance

All AEs will be recorded in the participants’ medical notes and on the eCRFs. AEs will be recorded from the day of randomization until the last visit or until withdrawal, with the exception of those considered related to the IMP, which will be followed until resolution, a stable outcome, or death. All AEs are assessed for severity, causality, expectedness, and seriousness by an investigator; all are reviewed by the Independent Data Monitoring and Ethics Committee (IDMEC). In accordance with current legislation, AEs will, where necessary, undergo expedited reporting to the sponsor and to the Medicines and Healthcare products Regulatory Agency (MHRA) within the required timelines.

### Statistical Considerations

#### Overview

A pragmatic decision was taken to recruit and analyze data on 90 patients: 45 in each group. This sample size is the minimum conventional threshold for making parameter estimates in pilot studies [33]. The parameter estimates in this trial will be used to inform a large, multicenter clinical trial. 

#### Data Management

Study data are recorded in each patient’s medical notes before being recorded onto eCRFs, which are developed and managed by the NCTU. The eCRF has been built using the Red Pill system supplied by Sealed Envelope Ltd. Data entered onto the eCRF must be consistent with the information in the medical notes. Patients are identified using a unique study ID; all data passed to the NCTU will have patient identifiers removed, with the exception of date of birth, gender, ethnicity, and study ID. Data cleaning is provided by staff within the NCTU.

#### Statistical Analysis

Primary analysis will follow intention-to-treat principles with patient data analyzed according to randomization and irrespective of intervention received; other analysis groups, such as per-protocol groups, may be considered subsequently. Every effort will be made to retain and include all patients who are part of the trial.

Data will be summarized by study group. Mean or median will summarize continuous variables, whereas number and percentage will be used to summarize categorical variables. A general linear model will be used to analyze the primary outcome considering all covariates used in the randomization scheme. Similar methods will be used to analyze all continuous secondary outcomes. Data of other types will be analyzed using generalized linear models with appropriate distributions.

The numbers of MACCE after 12 months will be compared between arms by the use of standardized rates (eg, by consideration of the number of events per patient month in each arm). Impact of missing data will be explored by tabulating the proportion of missing data in each arm. A full statistical analysis plan will be developed for the outcome measures and agreed upon with the IDMEC and the chief investigator prior to any analysis being undertaken.

### Trial Governance

The trial is sponsored by South Tees Hospitals National Health Service (NHS) Foundation Trust and funded by T.A. Sciences, Inc, New York, USA. The trial is being run in collaboration with the NCTU at Newcastle University. The sponsor and funder were not involved in the trial design. The sponsor has delegated the trial design; collection, management, analysis, and interpretation of data; report writing; and publications to the chief investigator and coinvestigators. The trial is overseen by the Trial Steering Committee, which meets every 6 months and includes an independent chair and two other independent members, one of whom is a patient. In addition, the trial includes the IDMEC, which meets every 6 months and oversees all ethical and safety issues in accordance with a study-specific DAMOCLES (Data Monitoring Committees: Lessons, Ethics, and Statistics) charter. All members are independent of the study team, although the trial manager, chief investigator, and some other members of the Trial Management Group attend the open sessions in order to inform the committee about the trial progress. The IDMEC makes recommendations to the Trial Steering Committee. The day-to-day supervision of the trial will be the responsibility of the Trial Management Group, who report to the Trial Steering Committee.

### Dissemination and Publications Policy

The trial will be published in peer-reviewed journals following the end of the trial, and the data will be presented at national and international meetings. We do not intend to use professional writers. Results of the trial will also be reported to the funder, sponsor, and the Research Ethics Committee within one year after the end of the trial. Trial participants will be informed about the trial results and their treatment allocation at the end of the trial, including a lay summary. The datasets analyzed during this study are available from the corresponding author on reasonable request.

## Results

The study received NHS ethics approval on August 9, 2018; MHRA approval on October 19, 2018; and NHS Health Research Authority approval on October 22, 2018. The trial began recruiting participants in January 2019 and completed recruitment in March 2020; the trial is due to report results in 2021.

During the COVID-19 pandemic, all patients will have their visits carried out remotely. Primary and secondary outcome measures that are not able to be carried out remotely will occur later than planned, on-site, as soon as it is safe for the patients to do so and when laboratories are able to process and analyze trial samples. Measures of blood pressure and blood glucose will be undertaken by patients who have not yet attended the site for their 6-month visit. Portable blood pressure monitors and blood glucose monitors will be sent to those patients to be used to record their results at home when required as per the schedule of events for the duration of the pandemic. Some patients may require continuation of the IMP beyond 12 months to enable them to attend the hospital for their final trial visit assessments. The 6-month and 12-month visits will occur as soon as it is safe for this group of patients to attend the hospital and when the trial laboratories are able to accommodate processing and analysis of trial samples.

## Discussion

ACS, the acute manifestation of atherosclerosis, is a leading cause of mortality and morbidity. Age is an independent risk factor for adverse cardiovascular outcomes after ACS, and this is likely related to upregulation of the inflammatory response that occurs with aging. Improvements in cardiovascular outcomes over the last two decades have been realized mainly in younger patients [34]. There remains an unmet need for novel therapeutic approaches in older patients following ACS [35,36].

The CANTOS (Canakinumab Anti-Inflammatory Thrombosis Outcomes Study) trial has provided strong evidence for the critical role of inflammation in atherosclerosis by showing that canakinumab—a human monoclonal anti-IL-1β antibody—led to a reduction in major cardiovascular events independent of lipid-level lowering [4]. Canakinumab was associated with a higher incidence of nonfatal infection, potentially limiting its use in older patients with CHD.

TA-65MD, the only available human telomerase activator, has been shown to increase TL in human subjects. We hypothesize that maintaining and/or lengthening telomeres can reduce T cell immunosenescence, thus reducing their proinflammatory effects. Telomerase has also been shown to have telomere-independent properties in vitro, namely, a protective effect in cells under oxidative stress and an ability to regulate FMD in the human microvasculature.

The proposed pilot trial in older patients with CHD will provide findings not previously investigated outside in vitro or preclinical models, which will substantially enhance our understanding of telomerase activators in this population and their potential for benefit. The double-blind, randomized, placebo-controlled design of the TACTIC trial will provide the gold standard for evaluating signs of efficacy of TA-65MD. Should the results indicate reduced frequency of immunosenescent CD8+ T cells and improvements in TL as well as endothelial function, we will aim to follow up with a larger, multicenter efficacy trial in patients to determine if TA-65MD is beneficial in the treatment of CHD.

## Abbreviations

ACS: acute coronary syndrome
AE: adverse event
CAD: coronary artery disease
CAG: cycloastragenol
CANTOS: Canakinumab Anti-Inflammatory Thrombosis Outcomes Study
CHD: coronary heart disease
CMV: cytomegalovirus
DAMOCLES: Data Monitoring Committees: Lessons, Ethics, and Statistics
eCRF: electronic case report form
EudraCT: European Union Drug Regulating Authorities Clinical Trials Database
FACS: fluorescence-activated cell sorting
flow-FISH: flow cytometry-fluorescent in situ hybridization
FMD: flow-mediated dilation
GCP: Good Clinical Practice
GRAS: Generally Recognized As Safe
hsCRP: high‐sensitivity C‐reactive protein
ICH: International Conference on Harmonisation
IDMEC: Independent Data Monitoring and Ethics Committee
IL-1: interleukin 1
IMP: investigational medicinal product
ISRCTN: International Standard Randomized Controlled Trial Number
MACCE: major adverse cardiac and cerebrovascular events
MHRA: Medicines and Healthcare products Regulatory Agency
MI: myocardial infarction
MRC-5: Medical Research Council cell strain-5
NCTU: Newcastle Clinical Trial Unit
NHS: National Health Service
NSTEMI: non-ST elevation myocardial infarction
NT-proBNP: N-terminal fragment of the prohormone brain-type natriuretic peptide
PAT: Peripheral Arterial Tone (in EndoPAT)
PBMC: peripheral blood mononuclear cell
PCR: polymerase chain reaction
STEMI: ST elevation myocardial infarction
TA: telomerase activity
TACTIC: Telomerase ACTivator to reverse Immunosenescence in Acute Coronary Syndrome
TBARS: thiobarbituric acid reactive substances
TEMRA: T effector memory cells re-expressing CD45RA (CD45 expressing exon A)
TERT: telomerase reverse transcriptase
TL: telomere length
TRAP: telomeric repeat amplification protocol
